# Metabolite Dysregulation by Pranlukast in *Mycobacterium tuberculosis*

**DOI:** 10.3390/molecules27051520

**Published:** 2022-02-24

**Authors:** Soujanya D. Yelamanchi, Sumaithangi Thattai Arun Kumar, Archita Mishra, Thottethodi Subrahmanya Keshava Prasad, Avadhesha Surolia

**Affiliations:** 1 Molecular Biophysics Unit, Indian Institute of Science, Bangalore 560 012, India; soujanyay@iisc.ac.in (S.D.Y.); mishra_archita@immunol.a-star.edu.sg (A.M.); 2 Center for Systems Biology and Molecular Medicine, Yenepoya Research Center, Yenepoya University, Mangalore 575 018, India; arunst@yenepoya.edu.in (S.T.A.K.); keshav@yenepoya.edu.in (T.S.K.P.)

**Keywords:** bacteria, antagonist, untargeted metabolomics, targeted metabolomics, mass spectrometer

## Abstract

*Mycobacterium tuberculosis* has been infecting millions of people worldwide over the years, causing tuberculosis. Drugs targeting distinct cellular mechanisms including synthesis of the cell wall, lipids, proteins, and nucleic acids in *Mtb* are currently being used for the treatment of TB. Although extensive research is being carried out at the molecular level in the infected host and pathogen, the identification of suitable drug targets and drugs remains under explored. Pranlukast, an allosteric inhibitor of *Mt*ArgJ (*Mtb* ornithine acetyltransferase) has previously been shown to inhibit the survival and virulence of *Mtb*. The main objective of this study was to identify the altered metabolic pathways and biological processes associated with the differentially expressed metabolites by PRK in *Mtb*. Here in this study, metabolomics was carried out using an LC-MS/MS-based approach. Collectively, 50 metabolites were identified to be differentially expressed with a significant *p*-value through a global metabolomic approach using a high-resolution mass spectrometer. Metabolites downstream of argJ were downregulated in the arginine biosynthetic pathway following pranlukast treatment. Predicted human protein interactors of pranlukast-treated *Mtb* metabolome were identified in association with autophagy, inflammation, DNA repair, and other immune-related processes. Further metabolites including *N*-acetylglutamate, argininosuccinate, L-arginine, succinate, ergothioneine, and L-phenylalanine were validated by multiple reaction monitoring, a targeted mass spectrometry-based metabolomic approach. This study facilitates the understanding of pranlukast-mediated metabolic changes in *Mtb* and holds the potential to identify novel therapeutic approaches using metabolic pathways in *Mtb*.

## 1. Introduction

*Mycobacterium tuberculosis* (*Mtb*), a devastating pathogen, majorly infects the lungs, causing tuberculosis (TB), which accounts for one of the top ten leading causes of death worldwide. Approximately, every year 10 million patients are infected annually with *Mtb* of which 1.4 million deaths are being reported according to the latest WHO 2019 report. Incidence of multidrug-resistant (MDR) and extremely drug-resistant (XDR) strains has become a major cause of concern in treating TB, where the success rate for treatment of MDR-TB was 57%, while XDR-TB was 39% and drug vulnerable TB was 85% (WHO, 2019) [1]. Various drugs that have been identified in the recent past, including bedaquiline, capreomycin, linezolid, and delamanid, inhibit energy metabolism, protein synthesis, and cell wall synthesis in *Mtb* [2]. The current anti-TB drugs exhibit several side effects on the host and have led to the emergence of drug-resistant genes in *Mtb* [3]. Consequently, the development of new anti-tubercular agents and scrutiny of their mechanism of action needs more emphasis.

Metabolic pathways unique to *Mtb* hold considerable potential for developing anti-tubercular agents. Among the various metabolic pathways, arginine biosynthesis is one of the indispensable pathways required for the virulence and survival of *Mtb* [4,5]. Thus, mutants of *argF* encoding ornithine carbamoyltransferase exhibit decreased pathogenicity and survival of *Mtb* in immunocompetent and immunocompromised mice [6]. Recently, *argB* mutants have shown decreased survival of *Mtb* by inducing oxidative stress [7,8]. While all the enzymes of the arginine pathway have been proposed to be essential for *Mtb* growth, among them *argJ* has been considered as a promising target for drug discovery and development against *Mtb* survival due to the lack of a homolog in humans [7,8]. The gene *argJ* encodes for the monofunctional enzyme ornithine acetyltransferase in *Mtb*, which acts on the substrates of first and fifth reactions in the arginine biosynthetic pathway [9]. Pranlukast (PRK), an antagonist of cysteinyl leukotriene receptor 1, is used in the treatment of asthma [10,11]. Recently, PRK has been shown to act as an allosteric inhibitor of *Mt*ArgJ, which in turn inhibits the survival of *Mtb* in cultures, a macrophage infection model, and in vivo in mice models of TB [12].

Metabolites that catalyze chemical reactions in distinct metabolic pathways play a significant role in regulating cellular functions through protein–metabolite interactions [13,14]. Mass spectrometry-based metabolomics is emerging with implications in the field of disease diagnosis, biomarker discovery, drug efficacy, and screening [15]. Because of the demonstrated ability of PRK to curtail the growth or pathogenesis of *Mtb*, understanding the global changes in the *Mtb* metabolome brought about by PRK is imperative for unravelling the in-depth mechanisms associated with its metabolism [16]. Therefore, in this study, untargeted metabolomics, followed by a targeted approach, was executed to identify the dysregulated metabolites by PRK in *Mtb*. To our knowledge, the dysregulation of metabolites associated with this drug in *Mtb* has not been studied so far. In this study, 30 metabolites in positive mode and 20 metabolites in negative mode were captured to be significantly dysregulated at MS2 level by PRK treatment. Pathway analysis showed significant enrichment of arginine and proline metabolic pathways. Further, downregulation of argJ downstream metabolites including N-acetylglutamate, argininosuccinate, and L-arginine, in addition to L-ergothioneine and L-phenylalanine, was validated in this study. Host protein target prediction against the dysregulated metabolome by PRK highlighted their association with inflammation, autophagy, phagocytosis, and other immune-related responses.

## 2. Results

### 2.1. Mass Spectrometry Analysis of PRK-Treated Mtb H37Rv

**Figure 1:**
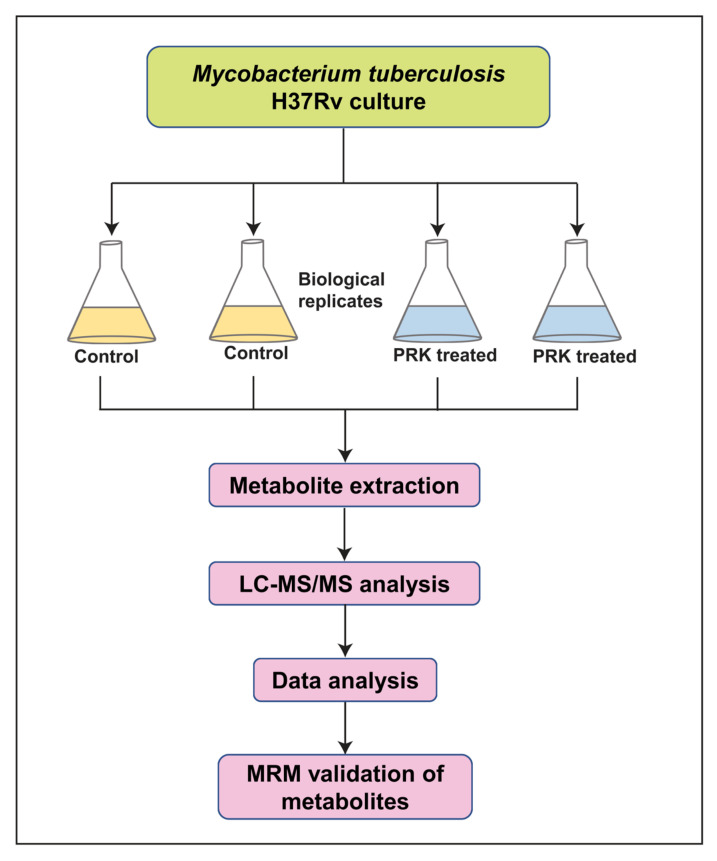
Schematic representation of the metabolomics experimental pipeline. The details of samples and the experimental pipeline employed for targeted and untargeted approach are illustrated.

**Figure 2:**
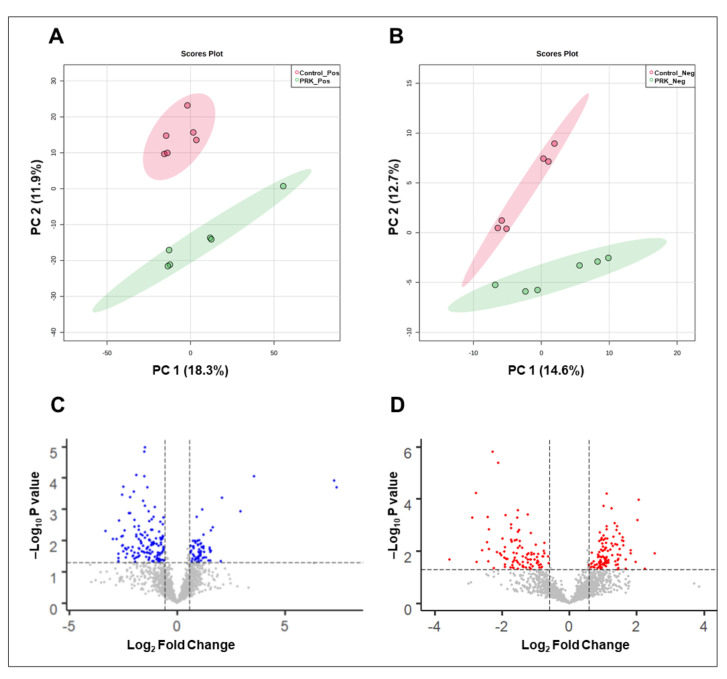
Data visualization plots. PCA clustering of PRK-treated and untreated *Mtb* H37Rv samples in (**A**) positive mode and (**B**) negative mode. Volcano plots showing the distribution of identified features in (**C**) positive mode and (**D**) negative mode. The differentially expressed metabolites with *p*-value ≤ 0.05 are highlighted in blue and red in the respective plots.

LC-MS/MS analysis of mycobacterial cells with and without PRK treatment was carried out in technical triplicates for each of the two biological replicates separately in both positive and negative modes. A schematic workflow deployed for mass spectrometry analysis is shown in Figure 1. The analysis led to the identification of 2330 aligned peak features in positive mode and 2084 aligned peak features in negative mode. A complete list of aligned peaks acquired from both positive and negative ion modes is provided in Supplementary Data, Tables S1 and S2, respectively. Of these, metabolite features expressed in at least two technical replicates per biological group were taken further for downstream analysis. These included 2264 features in positive mode and 2058 features in negative mode. A total of 426 metabolite features in positive mode and 219 metabolite features in the negative mode were assigned to *Mtb* H37Rv species in BioCyc and KEGG databases. Due to limitations in metabolite assignment, features lacking assignment at MS2 level were assigned at precursor level by mapping to their respective *m*/*z*. In positive mode 1335 aligned peaks and in negative mode 1058 aligned peaks were assigned at precursor level. Subsequently, unsupervised principal component analysis (PCA) on PRK-treated and untreated samples was performed to observe the clustering patterns between replicates and groups. The analysis resulted in close clustering among the replicates and distinct separation between the groups, as illustrated in Figure 2A,B.

The quality of the mass spectrometry data was analyzed by comparing blank runs with control and PRK sample runs in order to rule out any carryover of sample features [17]. Therefore, PCA analysis was performed, which showed distinct separation of blank runs from sample groups without leaving any carryover from samples. In addition, supervised PLS discriminant analysis (PLS-DA) with variable importance in projection scores showed the significant features were substantially unidentified or insignificant in blank runs [18]. PCA and PLS-DA illustrations in positive and negative modes are shown in Supplementary Data, Figure S1.

### 2.2. Differentially Expressed Mtb Metabolites by PRK

Fold change analysis was performed using a two-sample *t*-test to calculate the *p*-values. A total of 195 metabolite features in positive mode and 209 features in the negative mode were differentially expressed with a 1.5-fold change cut-off and *p*-value ≤ 0.05. Collectively, a non-redundant number of 78 metabolites in positive mode and 79 metabolites in negative mode assigned at MS1 level were found to be differentially expressed. Similarly, 30 metabolites in positive mode and 20 metabolites in negative mode assigned at MS2 level were differentially regulated. Volcano plots of the differentially expressed features are shown in Figure 2C,D. A partial list of the significantly dysregulated metabolites assigned at the MS2 level is provided in Table 1. A complete list of differentially expressed metabolites in positive mode and negative mode is provided in Supplementary Data, Tables S3 and S4, respectively.

### 2.3. Pathway Analysis and Metabolite Classification

**Figure 3:**
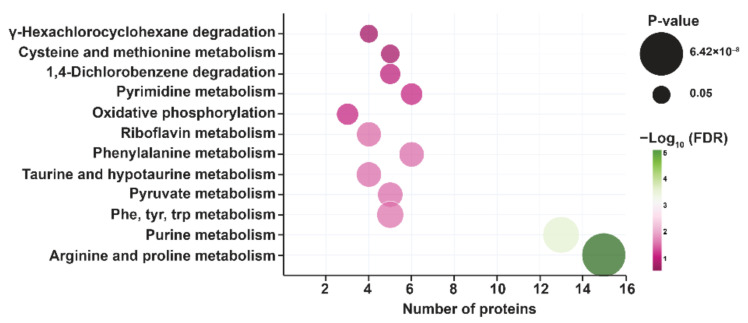
Pathway enrichment with significant *p*-value and FDR is shown as a bubble plot. The size of the bubble represents the *p*-value and color scale represents FDR for each pathway.

Pathway analysis of the differentially expressed metabolites was carried out against the *Mtb* H37Rv database to understand the changes in metabolic pathways in *Mtb* in response to PRK. Pathways including arginine and proline metabolism, purine, pyrimidine, and phenylalanine metabolic pathways along with others as shown in Figure 3 were significantly enriched with FDR ≤ 0.05. Interestingly, arginine and its downstream metabolites—L-agmatine, 4-guanidinobutanamide, and 2-oxoarginine—were downregulated in the arginine and proline metabolic pathway. Metabolite classification of the differentially expressed metabolites was performed using MBROLE against the *Mtb* H37Rv database. A portion of the differentially expressed metabolites by PRK was classified as nucleotides, amino acids, vitamins and cofactors, and fatty acids.

### 2.4. Host Protein Target Prediction against PRK-Treated Mtb-Dysregulated Metabolites

**Figure 4:**
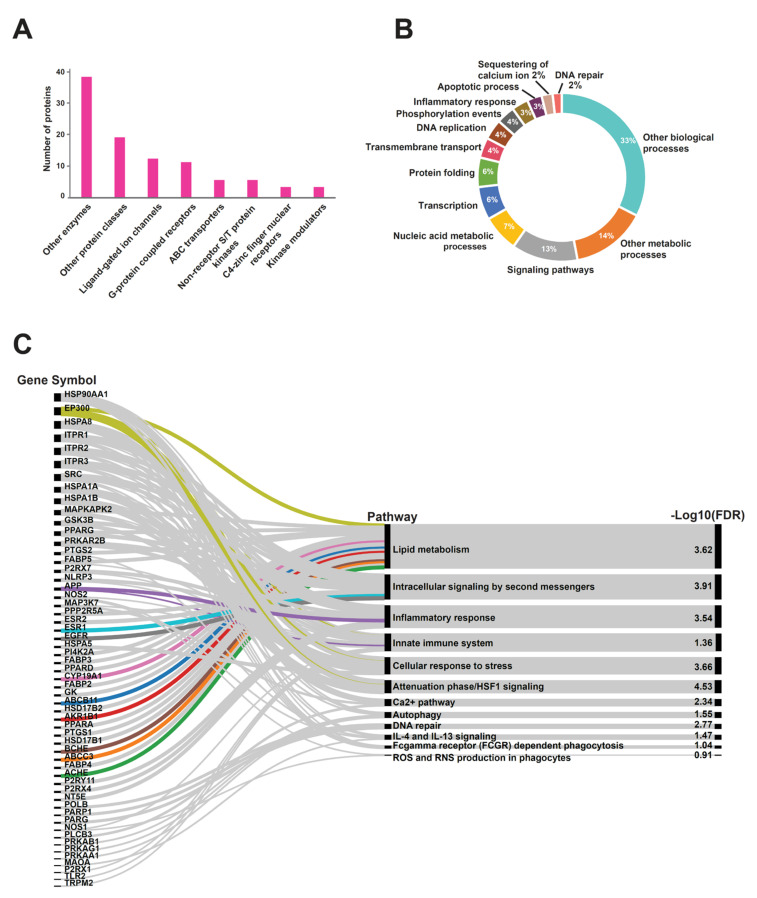
Gene Ontology classification of predicted protein targets—(**A**) protein classes and (**B**) biological processes. (**C**) Pathway enrichment showed with an alluvial diagram. The thickness of the correlation lines connecting genes to pathways represents a significant *p*-value (≤0.05).

Identification of predicted human protein targets against differentially expressed *Mtb* metabolome by PRK provides insights in understanding the host functional processes likely impacted in response to the combined effect of drug and *Mtb* infection. Therefore, human protein targets were analyzed in this study using a publicly accessible tool, BindingDB, which is a repository of protein–metabolite interactions essentially comprising experimentally proven data from the scientific literature [19]. In this study, only the significantly dysregulated metabolites with an assignment at MS2 level were chosen to obtain protein targets. The PubChem identifiers of these metabolites were converted to SMILES ID that served as an input source for BindingDB analysis. Collectively, 102 host protein targets were identified against 46 non-redundant *Mtb*-dysregulated metabolites with a similarity score ≥ 85% (Supplementary Data, Table S5). The protein targets were subjected to Gene Ontology (GO) analysis in order to understand their associated cellular processes and classification against PRK and *Mtb*. The predicted protein targets belonged to various classes including ligand-gated ion channels, G-protein-coupled receptors, ABC transporters, non-receptor S/T kinases, and other enzymes and protein classes (Figure 4A). These proteins were found to be involved in transcription, protein folding, transmembrane transport, inflammatory response, and sequestering of calcium ions, in addition to other biological processes (Figure 4B).

**Figure 5:**
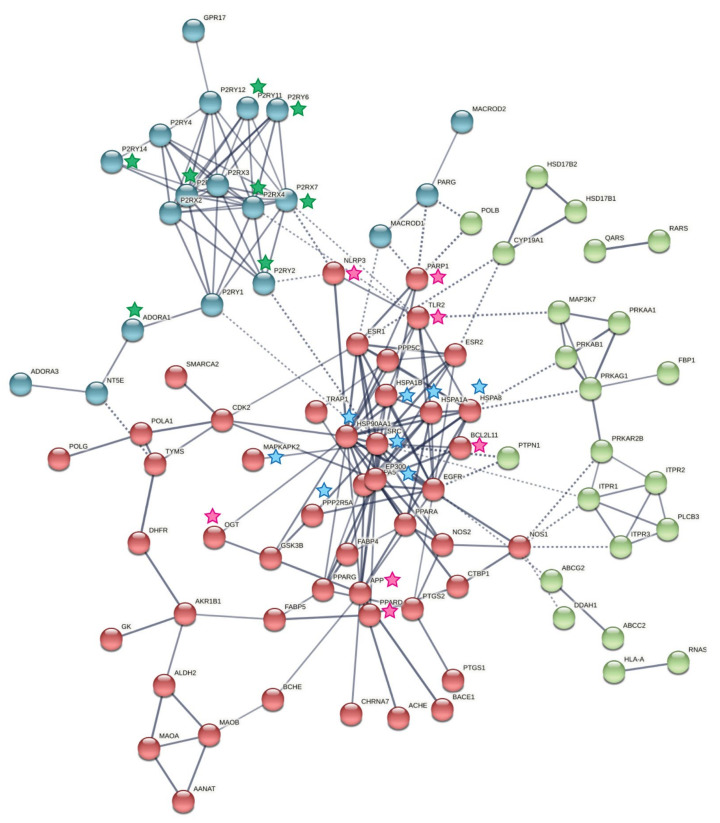
Interaction network map of protein targets of altered metabolites showing clustering of proteins into three groups. The edges connecting the nodes represent high confidence. Proteins associated with apoptosis and immune response are highlighted with pink and blue stars, respectively, while proteins associated with inflammation are highlighted with green stars.

Pathway analysis of the predicted protein targets was performed using the REACTOME pathway browser. Interestingly, pathways such as inflammatory response, autophagy, immune system, Fcγ receptor-mediated phagocytosis, and DNA repair were enriched with significant p-value and FDR (Figure 4C). Further, network analysis of these protein targets was grouped using a K-means clustering algorithm in the STRING web-based interface. Proteins involved in apoptosis (APP, NLRP3, OGT, TLR2, BCL2L11, PARP1, PPARD) and immune response (SRC, PPP2R5A HSPA8, HSP90AA1, HSPA1B, HSPA1A, MAPKAPK2, EP300) were clustered together in red nodes along with other proteins in Figure 5. In addition, close clustering of purinergic receptors such as P2RX1, P2RX4, P2RX7, P2RY2, P2RY6, P2RY11, P2RY14, and ADORA1 was observed that is known to be related to inflammation. These proteins are segmented as blue nodes in Figure 5.

### 2.5. Validation of Metabolites

**Figure 6:**
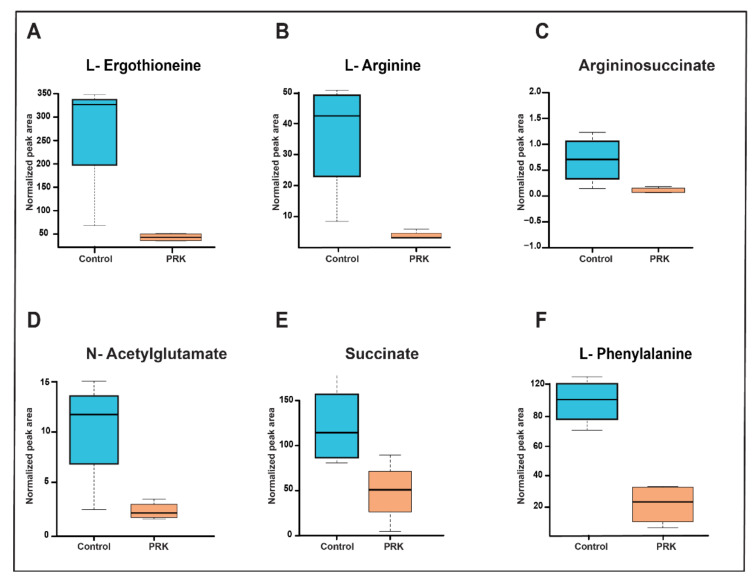
Box plots of validated metabolites showing differential expression of metabolites in control and PRK-treated *Mtb* H37Rv groups. (**A**) L-Ergothioneine, (**B**) L-Arginine (**C**) Argininosuccinate, (**D**) N-acetylglutamate, (**E**) Succinate, and (**F**) L-Phenylalanine.

**Figure 7:**
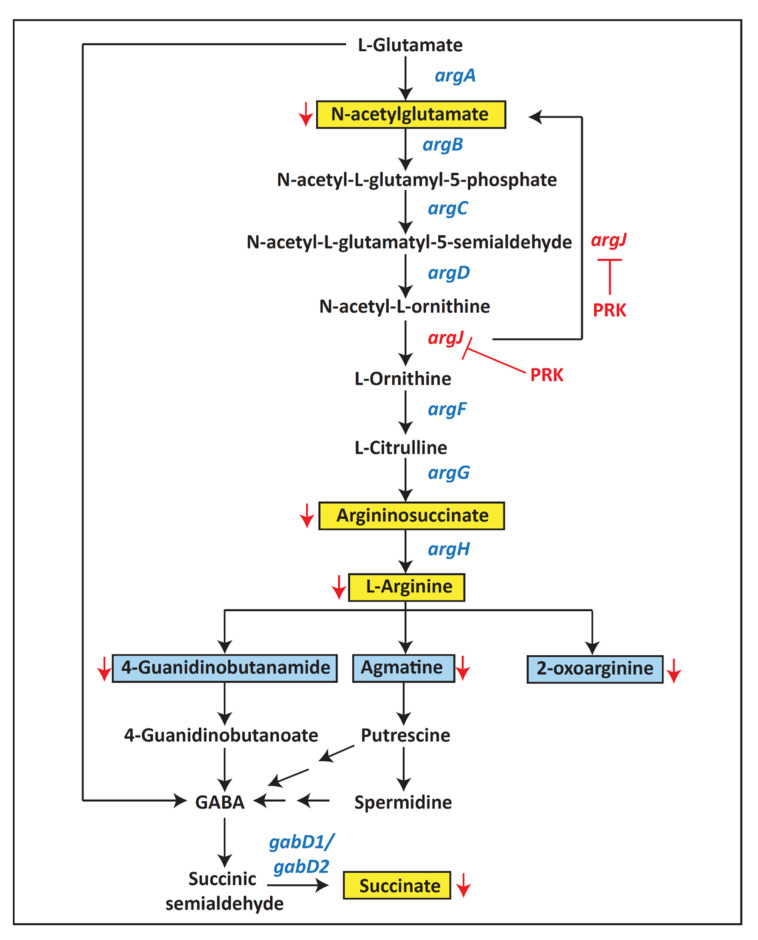
Pathway map of arginine metabolism and GABA shunt. MRM-validated metabolites are highlighted in yellow, while the metabolites dysregulated in the global analysis are highlighted in blue in the pathway.

A total of 20 metabolites that are involved in arginine metabolism, TCA cycle, and others including amino acids, purines, pyrimidines, and antioxidants were validated using the multiple reaction monitoring (MRM) approach. Of these, six metabolites, including L-arginine, *N*-acetylglutamate, ergothioneine, argininosuccinate, succinate, and L-phenylalanine, were significantly dysregulated with FC cut-off of 1.5 and *p-*value ≤ 0.05. A box plot of these metabolites is shown in Figure 6. Meanwhile, the remaining 14 validated metabolites were neither dysregulated nor had a significant p-value. The transition details and optimization parameters of all the 20 validated metabolites are provided in Supplementary Data, Table S6. A pathway map of arginine metabolism with a highlight of metabolites identified through targeted and global analysis at MS2 level is shown in Figure 7.

## 3. Discussion

The role of the arginine biosynthetic pathway in the survival and virulence of *Mtb* is currently receiving a lot of scrutiny. However, there are no drugs known so far to target enzymes of this pathway other than PRK and sorafenib (SRB), which target *Mt*ArgJ [20]. The absence of the *Mt*ArgJ homolog gene in humans and the vital requirement of this gene for *Mtb* growth and pathogenicity anoints *Mt*ArgJ as an important drug target against this devastating pathogen. Since PRK targets an allosteric site on *Mt*ArgJ, cross-reactivity with the molecules similar to substrate and additional off-target effects are likely eliminated. Moreover, the dearth of an equivalent gene in humans confers specific binding of the drugs, thus minimizing cross-reaction in *Mtb*-infected hosts. PRK has shown significant inhibition of *Mtb* survival under in vitro and in vivo conditions compared with SRB at same concentrations. Similarly, PRK in combination therapy with rifampicin and isoniazid reduced CFU to approximately 40- to 50-fold compared with SRB. Therefore, metabolomic analysis was carried out using PRK at a concentration of 1 µg/mL, which has been reported to barely inhibit the survival of *Mtb* [12].

A previous study has shown that downstream metabolites of argB and argF enzymes in the arginine biosynthetic pathway were depleted in the respective mutant strains of *Mtb*. In addition to these metabolites, antioxidants—ergothioneine and mycothiol—are downregulated in argB and argF mutants of *Mtb*. The authors also have shown that arginine deficiency augments DNA damage through ROS production, thus killing *Mtb* [7]. Further, L-ergothioneine is known to play vital roles in *Mtb*, where it confers defensive response against oxidative stress, anti-TB drugs, alkylating agents, and metals and also augments virulence of *Mtb* in the host [21]. Interestingly, decreased expression of ergothioneine and arginine pathway metabolites—*N*-acetylglutamate, argininosuccinate, and arginine—were observed in this study by a targeted approach. Therefore, it is predicted that PRK could have a similar role in sterilizing *Mtb*. However, further experiments are required to confirm the mechanism of action of *Mtb* killing by PRK.

Succinate, an intermediate metabolite of GABA shunt or TCA cycle, is a substrate of succinate dehydrogenase enzyme in the electron transport chain (ETC) [22]. Oxidation of succinate to fumarate is known to enhance menaquinol levels that pump protons to complex III and IV, thus generating ATP synthesis and membrane potential [23]. Decreased levels of succinate by PRK may pose dysregulation of the ETC pathway, as it correlated with decreased menaquinol and ATP levels in this study. ATP is an essential metabolite for various metabolic processes in *Mtb*. Bedaquiline, a well-known anti-tubercular drug, inhibits ATP synthase that leads to depletion of ATP and disruption of pH homeostasis, thus affecting bacterial survival [24,25]. CTP and CDP are also metabolite co-substrates of phosphatidylinositol biosynthesis and the non-mevalonate pathway [26,27]. CTP and CDP were downregulated by PRK. Further, 2-C-methyl-D-erythritol-2,4-cyclodiphosphate (MEcPP), a metabolite of the non-mevalonate pathway of isoprenoid biosynthesis, is synthesized by the activity of MEcPP synthase (ispF). Knockout studies have shown that the ispF gene is essential for the survival of *Mtb* [28]. Moreover, the lipophilic nature of the ispF enzyme active site is considered to be the best target for drugs compared with other enzymes of the non-mevalonate pathway [29,30]. MEcPP, an anti-stressor molecule, is known to increase in the presence of oxidative stress in various bacterial species [31]. Interestingly, MEcPP was observed to be 0.5-fold downregulated by PRK.

Phenylalanine catabolizes to acetyl CoA and succinyl CoA through the formation of phenylacetic acid in bacteria [32]. Phenylalanine was 0.44-fold downregulated in MRM-based experiments. α-Ketoglutarate synthesized from oxalosuccinate enters into the arginine biosynthetic pathway through the formation of glutamate. TCA cycle metabolite oxalosuccinate was upregulated in this study. The pentose phosphate pathway end product enters into purine, pyrimidine metabolism for the synthesis of nucleotides. In this study, the central metabolite of the pentose phosphate pathway, 6-phospho-D-gluconate, was observed to be downregulated.

Identification of predicted host protein targets against the PRK-regulated *Mtb* metabolome revealed interesting observations. The targeted proteins were identified in association with the pathogen–host interaction pathway. Previous studies have shown that PRK induces autophagy in the fibroblasts of mucopolysaccharidosis type IVA disease [33]. PRK is an antagonist of cysteinyl leukotriene receptor (CysLTR) that is widely expressed on innate immune cells, including macrophages. PRK is previously known to exhibit anti-inflammation by alleviating enzymes of leukotrienes and prostaglandin biosynthesis [34]. Leukotrienes that mediate inflammatory response bind to CysLTR [35]. Further, downregulation of lipid inflammatory molecules such as PTGS2, along with ALOX5 and ALOX5AP, associated with arachidonic acid metabolism by PRK in *Mtb*-infected Raw264.7 murine macrophages [12]. The predicted protein targets—PTGS1, PTGS2—were enriched in the lipid metabolic pathway, in addition to other fatty acid metabolic enzymes or proteins. Further studies on other protein targets related to lipid metabolism will provide better understanding of PRK-mediated regulation in an *Mtb*-infected host.

Network analysis showed close clustering of purinergic receptors that play a role in host inflammatory response. P2RX7 and ATP signaling induce necrosis and cell death through the production of pro-inflammatory cytokines upon *Mtb* infection [36]. Stimulation of P2RY6 induces the production of pro-inflammatory cytokines such as IL-8 in human monocytes treated with lipopolysaccharide (LPS) [37]. Further, UDP-P2RY6 signaling re-establishes the differentiation of monocytes through autophagy induction in CMML patients [38]. P2RY2 and P2RY14 receptors are pro-inflammatory, while P2RY11 are anti-inflammatory molecules [39]. In addition to purinergic receptors, clustering of proteins associated with apoptosis was also observed. Previous reports in *Mtb*-infected macrophage cell lines have shown overexpression of BCL2L11 and induction of apoptosis, indicating the involvement of BCL2L11 in the pro-apoptotic mechanism [40]. TLR2 gene deletion and anti-TLR2 antibodies studies have shown that signaling of TLR2 by cell wall protein Rv1016c induces apoptosis of *Mtb*-infected macrophages [41]. Further, NLRP3 is associated with pyroptosis in *Mtb*-infected macrophages [42]. In our previous study, PRK has been shown to inhibit apoptosis by alleviating pro-apoptotic signaling protein caspase-3 in *Mtb*-infected murine and human macrophage cell lines [12]. In addition, PRK has also been reported to exhibit protective effects by producing anti-inflammatory signaling molecules in ischemic brain-injured rats [43,44]. An association of PRK treatment with autophagy, apoptosis, and inflammation highlighted here serves to provide an impetus for further investigations in this context.

In the present study, mass spectrometry data were acquired at the MS2 level, and targeted analysis was performed on a couple of significant metabolites that are involved in the arginine biosynthetic pathway. In recent times, metabolomic analysis has been rapidly progressing from the modest approach of deploying quality control (QC) methods that are based on the comparison of blank profiles to the advent of pooled QC samples in multiple injections, along with sample data acquisition batches [17]. The concept of QC was introduced at the time the data acquisition was executed for this study [45]. Therefore, comparison of blank runs to the sample runs was carried out that did not result in any carryover of the sample metabolites, as reported in this study (See Supplementary Data, Figure S1 for details). Moreover, high-confidence data were generated, as the MS2 acquired features were assigned at MS1 and MS2 levels using the in-house MS2Compound tool [46]. Further, the data were predominantly dealt at the MS2 level in both untargeted and targeted approaches, and most of the metabolites were associated with arginine and proline biosynthetic pathways, along with ETC and purine metabolism. The study not only validates the mode of anti-tubercular action of PRK through targeting of *Mt*ArgJ but also provides a comprehensive insight into the altered levels of metabolites associated with *Mtb*-killing by PRK.

## 4. Materials and Methods

### 4.1. Mtb H37Rv Culture and Treatment Conditions

*Mtb* bacterial cultures were grown in 100 mL Middlebrook 7H9 media supplemented with 10% OADC and Tween-80 until the cultures reached the logarithmic phase of 0.6 OD. Subsequently, the bacterial cells were treated with the drug PRK (Tocris Bioscience, Bristol, UK) at a concentration of 1 µg/mL, and the control cells were treated with DMSO and incubated at 37 °C for 12 h. Preceding the bacterial cell harvest, the cell density was optimized to 0.4 OD in both DMSO and PRK-treated cultures (0.4 OD is ~0.6 × 10^8^ mycobacterial cells). The experiment was carried out in biological duplicates for both the conditions.

### 4.2. Mtb H37Rv Cell Llysis and Metabolite Extraction

The cultures were subjected to centrifugation at 5000 rpm for 10 min at 4 °C, and the pellets were washed with ice-cold PBS three times in order to remove the leftover media and the chemicals. The pellets were snap-frozen in liquid nitrogen and were resuspended in 1 mL of precooled resuspension buffer containing acetonitrile (ACN), methanol, and water at a ratio of 2:2:1. The cells were then subjected to mechanical cell lysis using 0.1 mm zirconia beads in a tissue homogenizer with a pulse of 6 m/s for 45 s each round. The process was repeated for four rounds by placing the tubes on the ice at regular intervals. The lysates were centrifuged at 14,000 rpm for 20 min at 4 °C, and the supernatants were separated and filtered using 0.22 µm filters (Corning). The samples were dried using a speed vacuum before LC-MS/MS analysis.

### 4.3. LC-MS/MS Analysis for Global Metabolomic Profiling

Samples were analyzed on an ABSciex QTRAP 6500 mass spectrometer (SCIEX, Framingham, MA, USA) in triplicates for each biological replicate in both positive and negative modes, respectively. The mass spectrometer was coupled with an Agilent 1290 Infinity II liquid chromatography system, C_18_ RRHD Zorbax column (20 × 150 mm, 1.8 μm particle size). The parameters for mass spectrometry analysis were set on Analyst software (version 1.6.3) with the inbuilt Analyst Device Drive. The metabolite separation was carried out using a 30 min LC method with 0.1% formic acid (solvent A) and 0.1% formic acid in 90% ACN (solvent B), and the flow rate was set to 0.3 mL/min. The LC method was set with gradient as 2.0% B for 1 min, 2.0–30% B for 9 min, 30–60% B for 7min, 60–95% B for 9 min, and 2% B for 4 min and with a flow rate of 0.300 mL/min. The sample injection volume was set as 15 µL per injection. The ABSciex QTRAP 6500 (triple quadrupole-linear ion trap) mass spectrometer uses the information dependent acquisition (IDA) method, which is built with an enhanced mass spectra (EMS) survey scan to identify the top five ions based on intensity in each scan, which are taken forward for tandem MS-enhanced product ion (EPI) scan. The EMS survey scan rapidly screens for all the compounds present in the sample. The IDA criteria were set to trigger dependent scans, while the EPI scans rapidly collected high-quality MS/MS data. For untargeted metabolomics, a general unknown screening (with EMS) can detect maximum compounds and metabolites.

The data acquisition was executed with the IDA method at low mass mode. The top five intense spectra from EMS mode were selected for analysis in EPI (MS2) mode, using high energy collisional-induced dissociation (CID). The default option, three mass windows per scan with respective scan times in QTRAP-6500, was selected. Further, metabolite data were acquired in both polarities at 4500 V in positive mode and at −4500 V in negative mode, with a probe temperature set to 450 °C. The compound parameters, including declustering potential (DP), were set to 75 V and collision energy (CE) was set to 45 V. The cycle time was set at 2.091 s per cycle. The MS2 data were acquired for biological duplicates and technical triplicates. In between every technical triplicate sample run, intermediate blank runs were executed on a mass spectrometer to avoid sample carryover between adjacent sample runs.

### 4.4. MZmine Data Analysis and Metabolite Assignment

The metabolite data analysis was carried out with MZmine version 2.53 [47]. The wiff files from Analyst software were converted to mzML files using the ProteoWizard MS Convert tool. The .mzML files of control and PRK-treated were analyzed for mass detection using the centroid mode with peak intensities set to a minimum of 1.0E3 at MS1 level and 1.0E1 at MS2 level. The *m*/*z* feature list was built by selecting the precursors comprising MS2-level information using the MS/MS peak list-builder algorithm. Features were then detected with the Peak extender algorithm with *m*/*z* tolerance of 0.05 Da. Chromatogram deconvolution with Noise Amplitude algorithm was selected, where a minimum peak height of 1.0E3, the noise peak height of 1.5E2, and retention time (RT) of 1 min and *m*/*z* tolerance of 0.1 Da for MS2 pairing was set for deconvolution of the feature list. Isotopic peak grouping with *m*/*z* tolerance of 0.25 Da, maximum charge of 4, and RT tolerance of 0.2 min were chosen. The deisotoped features were aligned with *m*/*z* tolerance of 50 ppm and *m*/*z* weight of 70%, along with RT tolerance of 0.5 min, RT threshold of 30% using the Join-Aligner algorithm. Further, gap filling was performed with the Peak finder (multi-threaded) algorithm with *m*/*z* tolerance and RT tolerance set to 0.05 Da and 0.6 min, respectively. A duplicate filter algorithm was applied with New Average filter mode to remove the duplicate peaks. Subsequently, the results containing feature ID, *m*/*z*, RT, and peak areas at the MS2 level were exported as .csv files. Similarly, MS2 information of MS1 masses was exported as .mgf files for metabolite assignment and further downstream analysis. Raw files including blank runs, PRK-treated and control sample runs were analyzed on MZmine using similar parameters that were employed for sample group analysis.

The .mgf files comprising MS1 and MS2 information were used to fetch metabolite details at the MS1 and MS2 level through the in-house inbuilt MS2Compound tool [46]. The metabolites of *Mtb* H37Rv from BioCyc and KEGG databases were computationally fragmented by using metabolite SMILES ID as input in the Competitive Fragmentation Modeling-ID (CFM-ID) tool [48]. Such fragmented details were used as the theoretical database for searching *Mtb* H37Rv metabolites. Parameters including precursor tolerance of 0.05 Da, a fragment tolerance of 0.5 Da, and a minimum of two fragment matches were set for searching the metabolites against the *Mtb* H37Rv database. The metabolites with rank 1 and the highest mS score were selected. Further, *m*/*z* features lacking metabolite assignment at the MS2 level were assigned at the MS1 level.

### 4.5. Statistical and Functional Analysis

Statistical analysis was carried out using the MetaboAnalyst version 5.0 [49] online tool. Fold changes were calculated from median normalized data. PCA analysis was employed with log_10_ data transformation and auto-scaling for positive mode data and mean centering for negative mode data. Metabolite classification and pathway analysis for the differentially expressed metabolites was performed against *Mtb* H37Rv species using MBROLE version 2.0 [50]. Protein interactors for the identified metabolites were predicted using the BindingDB database, and the protein clustering was carried out by using the STRING K-means algorithm with the confidence set to 0.7 for the interaction network. GO terms including biological processes and protein classes for predicted proteins were acquired against the *Homo sapiens* database from PANTHER version 16.0. Further, pathway analysis for the predicted proteins was executed using REACTOME.

### 4.6. Targeted Analysis of Metabolites by Multiple Reaction Monitoring (MRM)

The validation of the 20 metabolites was carried out with standards where each and every metabolite was individually optimized for LC and MS/MS parameters to acquire the RT and *m*/*z* transitions for precursor and product ions in addition to DP, EP, CE, and CXP. Samples for MRM analysis were carried out in technical duplicates for each of the two biological replicates using an ABSciex QTRAP 6500 mass spectrometer interfaced with a 1290 Infinity II HPLC system (Agilent Technologies, Santa Clara, CA, USA). The samples were injected onto the Zorbax RHP column with the dimensions of 2.1 mm × 150 mm, 2.7 μm (Agilent Technologies, USA) through the programmed autosampler. Metabolite separation was carried out using 0.1% formic acid in water (Solvent A) and 0.1% formic acid in 90% ACN (Solvent B). LC method was set with gradient as 2.0% B for 3 min, 2.0–10% B for 2 min, 10–30% B for 2 min, 30–70% B for 7 min, 70–98% B for 9 min, 2% B for 7 min, and with a flow rate of 0.300 mL/min. The total run time was 35 min, and 15 μL of the sample was injected into the column. Data were acquired in positive and negative modes depending on the property of the metabolite using MRM scan mode. The Analyst software, version 1.6.2 (AB SCIEX, Concord, Canada), was used to acquire data. Samples were ionized using the ESI source. Ion Source Gas 1 (GS1) at 25 psi, Ion Source Gas 2 (GS2) at 5.0 psi, Curtain gas (CUR) at 20.0 psi, ESI Source temperature at 450 °C, and Collision-activated dissociation (CAD) gas at medium were maintained. The ion spray voltage was set to 5500 V. The entire MS parameters, including RT, *m*/*z*, and ion intensities, were acquired through the Analyst software, and the data were extracted using Skyline [51]. The resulting MS data were assembled into a matrix. DP, CE, and CXP for Q1 and Q3 masses for the selected molecules are provided in Supplementary Data, Table S6. A total of 20 metabolites were detected and quantified (relative) in both positive and negative modes.

### 4.7. Data Availability

Mass spectrometry-derived metabolomic data were submitted to MetaboLights [52], which is a repository of metabolomic experiments including spectra, structures, and biological roles. The study details and data are available with the identifier MTBLS3465.

## 5. Conclusions

In this study, metabolomic profiling of differentially expressed metabolites was carried out using untargeted and targeted mass spectrometry-based approaches. Untargeted analysis carried out at the MS2 level enhances the confidence of metabolite assignment and its succeeding downstream analysis. In addition to metabolites associated with arginine and proline metabolism, metabolites associated with the electron transport chain were also identified in this study. Further studies are required to confirm the association of predicted protein targets and their related biological processes and pathways in *Mtb*-infected hosts. This study provides a framework or basis for the biological interpretation of metabolomic changes mediated by PRK in the pathogen.

## Figures and Tables

**Table 1:** A partial list of differentially expressed metabolites.

S. No	Metabolite	Mode of Acquisition	Fold Change	*p*-Value
1	L-Arginine	Positive	0.15	0.03
2	Agmatine	Negative	0.47	0.04
3	4-Guanidinobutanamide	Positive	0.25	0.04
4	2-Oxoarginine	Positive	0.15	0.04
5	5-Amino-6-(5′-phospho-D-ribitylamino) uracil	Positive	1.92	0.05
6	2-Methylmalate	Negative	1.59	0.03
7	S-methyl-5-thio-D-ribose	Positive	0.59	0.04
8	Oxalosuccinate	Positive	1.92	0.04
9	3-Hydroxypropionyl-CoA	Positive	0.61	0.03
10	Menaquinol	Positive	0.17	0.01
11	NADP	Positive	0.65	0.02
12	5′-Adenylyl sulfate	Positive	4.24	0.00
13	6-Phospho-D-gluconate	Positive	0.18	0.00
14	Cyclic-AMP	Negative	2.05	0.03
15	Inositol 1-phosphate	Negative	2.25	0.02

## Data Availability

Metabolomics data of this study is available at the MetaboLights database with the study identifier MTBLS3465.

## Supplementary Materials

Figure S1: Blank and sample group analysis by PCA analysis in (A) positive mode and (B) negative mode. Important features detected by PLS-DA analysis in (C) positive mode and (D) negative mode are shown, Table S1: A complete list of MS2 features identified in positive mode. The table contains headers of adducts, *m*/*z*, RT, metabolite names, MS2Compound score, and peak areas for the identified features. Identifiers including KEGG or BioCyc, PubChem, and HMDB are also appended, Table S2: A complete list of MS2 features identified in negative mode. The table contains headers of adducts, *m*/*z*, RT, metabolite names, MS2Compound score, and peak areas for the identified features. Identifiers including KEGG or BioCyc, PubChem, and HMDB are also appended, Table S3: List of differentially expressed metabolite features in positive mode. The table shows the details of RT, *m*/*z*, metabolite names, and assignment level for the significant (*p*-value ≤ 0.05) differentially expressed features in positive mode, Table S4: List of differentially expressed metabolite features in negative mode. The table shows the details of RT, *m*/*z*, metabolite names, and assignment level for the significant (*p*-value ≤ 0.05) differentially expressed features in negative mode, Table S5: Predicted protein targets for the differentially expressed metabolites are tabulated, Table S6: List of MRM-validated metabolites. The table shows the details of transitions and optimization parameters of MRM-validated metabolites.

## Sample Availability

Samples of the compounds are not available from the authors.

## Abbreviations

TB	Tuberculosis
PRK	Pranlukast
SRB	Sorafenib
PCA	Principal component analysis
PLS-DA	PLS discriminant analysis
GO	Gene Ontology
ACN	Acetonitrile
ETC	Electron transport chain
LPS	Lipopolysaccharide
MRM	Multiple reaction monitoring
QC	Quality control
FC	Fold change
RT	Retention time
DP	Declustering potential
EP	Entry potential
CE	Collision energy
CXP	Cell exit potential
