# Supplementary figures and images for: Metabolite Dysregulation by Pranlukast in Mycobacterium tuberculosis
Source: Molecules. 2022 Feb 24;27(5):1520. doi: 10.3390/molecules27051520 (PMC8911922; doi:10.3390/molecules27051520)

# Figure S1

## A

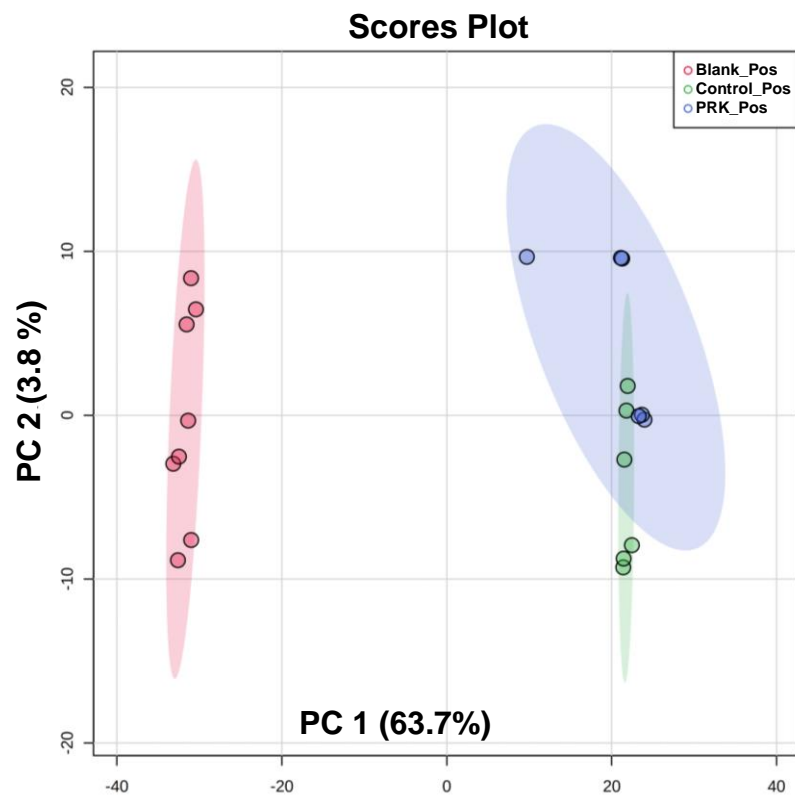

## C

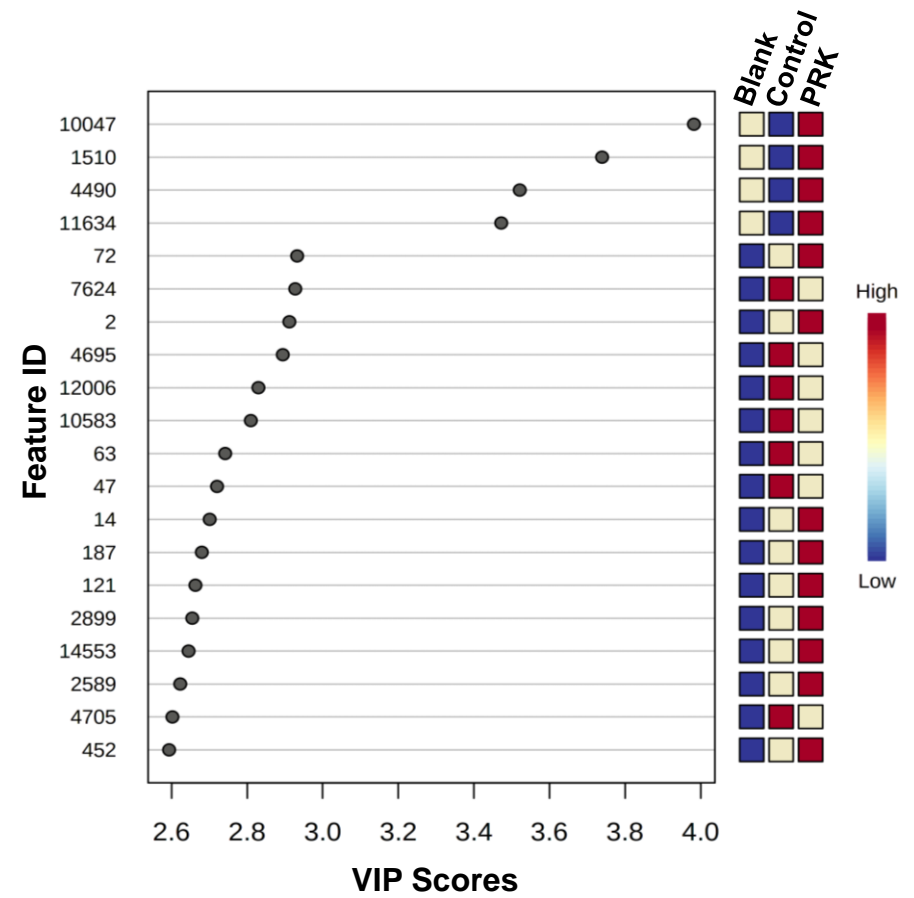

## B

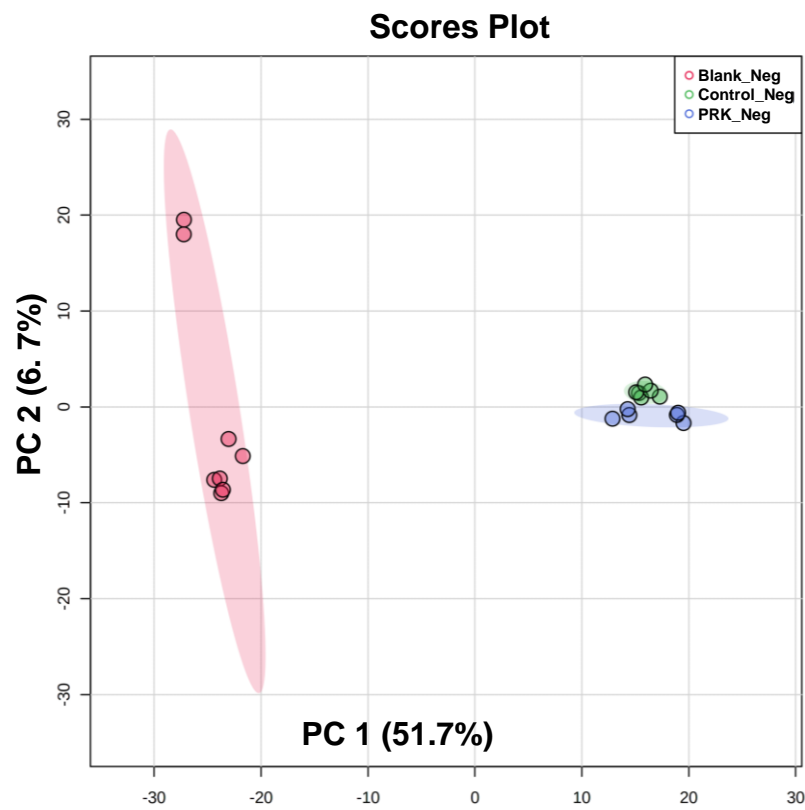

## D

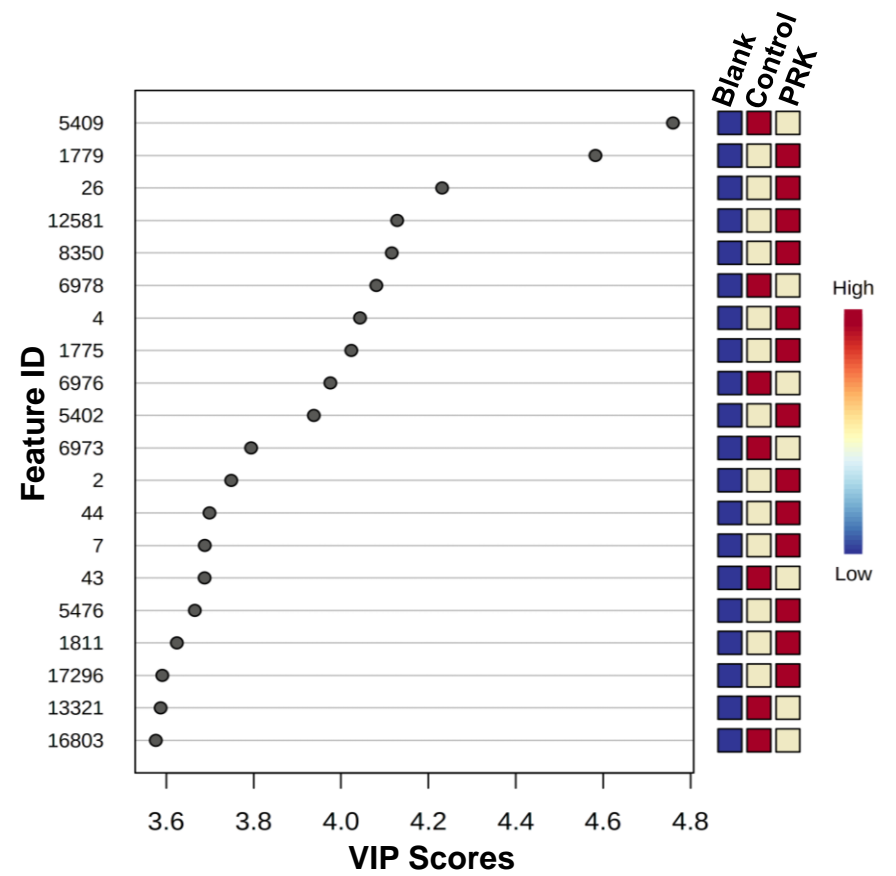

Supplement: Supplementary file 1 [file molecules-27-01520-s001.zip › Supplementary FigureS1_Yelamanchi_221221.pdf]
